# Mechanical analysis of avian feet: multiarticular muscles in grasping and perching

**DOI:** 10.1098/rsos.140350

**Published:** 2015-02-25

**Authors:** Spencer B. Backus, Diego Sustaita, Lael U. Odhner, Aaron M. Dollar

**Affiliations:** 1 Department of Mechanical Engineering and Materials Science, Yale University, New Haven, CT 06511, USA; 2 Department of Ecology and Evolutionary Biology, Brown University, Providence, RI 02917, USA

**Keywords:** birds, grasping, foot morphology, underactuated mechanisms, digit flexors

## Abstract

The grasping capability of birds' feet is a hallmark of their evolution, but the mechanics of avian foot function are not well understood. Two evolutionary trends that contribute to the mechanical complexity of the avian foot are the variation in the relative lengths of the phalanges and the subdivision and variation of the digital flexor musculature observed among taxa. We modelled the grasping behaviour of a simplified bird foot in response to the downward and upward forces imparted by carrying and perching tasks, respectively. Specifically, we compared the performance of various foot geometries performing these tasks when actuated by distally inserted flexors only, versus by both distally inserted and proximally inserted flexors. Our analysis demonstrates that most species possess relative phalanx lengths that are conducive to grasps actuated only by a single distally inserted tendon per digit. Furthermore, proximally inserted flexors are often required during perching, but the distally inserted flexors are sufficient when grasping and carrying objects. These results are reflected in differences in the relative development of proximally and distally inserted digital flexor musculature among ‘perching’ and ‘grasping’ taxa. Thus, our results shed light on the relative roles of variation in phalanx length and digit flexor muscle distribution in an integrative, mechanical context.

## 2. Introduction

Next to flight, a foot capable of grasping is a quintessential, but comparatively less appreciated and understudied, aspect of avian evolution [1–5]. Although it has been studied mostly in the context of perching, grasping plays essential roles in arboreal locomotion and feeding in many avian taxa (reviewed by Sustaita *et al.* [6]). The vast majority of bird species appear to have relatively less musculoskeletal mass allocated to the distal hind limbs compared with the grasping appendages of similarly sized mammals, yet many retain extremely dexterous multi-digit grasping and manipulation capabilities [6]. For example, in the passeriform foot, the number of phalanges (14) is high relative to the number (and sizes) of muscles acting on them—an atypical state in the grasping limbs of vertebrate tetrapods [6]. In addition, the variation in relative phalanx lengths observed among terrestrial and arboreal taxa [2,7] may affect the distribution of tendon forces across phalanges [8,9]. We believe that the multiarticular digit flexor muscle–tendon units in many bird species may be adaptive for allowing powerful grasping (e.g. for perching or prey prehension), with relatively few muscles and potentially lower musculoskeletal mass requirements.

In this paper, we apply analytical techniques used to examine robotic ‘hand’ functionality to simulate the behaviour of a prototypical bird foot in response to the forces imparted by carrying and perching tasks. We use our simulations to test the hypothesis that, for the range of phalanx lengths and object sizes observed in nature, a foot with a single distally inserted tendon per digit (hereafter referred to as the ‘single-tendon’ case) can perform as well as a foot with one tendon inserted distal to each joint (hereafter referred to as the ‘fully actuated’ case) for a given task. We begin with an in-depth discussion of the relevant background in avian foot morphology as it relates to grasping and perching functionality, followed by an overview of the mechanical concepts and mechanism types used in our analysis (§3), as well as the details of our model and simulations (§4). Lastly, we present the results from the simulation-based study (§5), and discuss the implications of those results in the context of avian anatomy and compare them with anatomical data from the literature (§6).

## 3. Background

### 3.1 Avian foot morphology and grasping

The behavioural and functional performance attributes of grasping have primarily been investigated in raptorial birds, such as hawks, falcons and owls [10–14], and to some extent in parrots [15,16]. These groups are highly specialized for powerful (raptors) and dexterous (parrots) manipulation with their feet, and these attributes are reflected in various aspects of their foot anatomy. For instance, raptor digital flexor muscle forces scale with positive allometry [12,17], and the sizes and shapes of their enlarged claws are correlated with prey immobilization [18] and feeding [19] behaviour. Parrots, on the other hand, demonstrate additional, and/or relatively greater development of, tarsal and digital extensor muscles that may afford them greater pedal dexterity [20,21]. Despite their taxonomic moniker, the ‘perching birds’ (Passeriformes) are in many respects comparatively less specialized for generating particularly powerful or dexterous grasps. Nevertheless, passeriforms have evolved a diversity of foot musculoskeletal configurations presumably adapted to various locomotor modes and substrates, with comparatively fewer muscles [22].

Among avian taxa, the number of toe actuators (i.e. flexor and extensor muscles) varies from 11 to 18: nine extrinsic muscles (five proximally inserted (‘superficial’) flexors, two distally inserted (‘deep’) flexors and two extensors arising from the tibiotarsus) and two to nine intrinsic muscles (adductors, abductors, and flexor and extensor brevia arising from the tarsometatarsus; based on Raikow [22]). However, there are far fewer muscle actuators than the number of joint motions that the foot is capable of performing. Most bird feet can execute between 28 and 32 joint motions (depending on the presence of a functional hallux) corresponding to flexion and extension of each of the 12–14 tarsometatarso-phalangeal and interphalangeal joints of digits I–IV, and abduction and adduction of the tarsometatarso-phalangeal joint for digits II and IV. Thus, depending on the taxon, the ratio of muscle actuators to directions of motion can vary from 11 : 32 (e.g. most perching birds (Passeriformes [23])) to 18 : 28 (e.g. emu (Dromaiidae)), and possibly greater when considering species for which data is not readily available.

**Figure 1.**
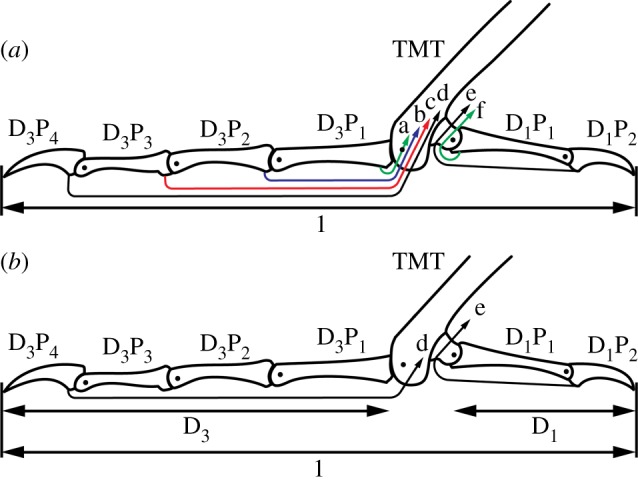
Diagram of the simplified bird foot geometry showing the tarsometatarsus (TMT), digits I (D_1_) and III (D_3_), their corresponding phalanges (P_1_–P_4_), and tendon insertions for the fully actuated (*a*) and single-tendon (*b*) models. Tendon insertions for the proximal (a; green) and distal (b; blue) branches of M. flexor perforatus digiti III, M. flexor perforans et perforatus digiti III (c; red), M. flexor digitorum longus (d; black), M. flexor hallucis longus (e; black), and M. flexor hallucis brevis (f; green) are shown. To eliminate the effect of variations in foot size among specimens, all geometries were normalized by the total span of the foot (1=*D*_1_*P*_1_+*D*_1_*P*_2_+distance between tarsometatarso-phalangeal joints+*D*_3_*P*_1_+*D*_3_*P*_2_+*D*_3_*P*_3_+*D*_3_*P*_4_).

The relationship between the number of independent muscle actuators and degrees of freedom (DOF) of the foot is further complicated by the complex tendon structures and their insertions on various digits. The tendons of the deep digital flexors (M. flexor digitorum longus and M. flexor hallucis longus) that insert on the distal ungual (claw-bearing) phalanges tend to be interconnected in various ways that couple their actions across the toes, particularly among digits II–IV [22]. The superficial flexors (M. flexor perforatus digiti II, M. flexor perforans et perforatus digiti II, M. flexor perforatus digiti III, M. flexor perforans et perforatus digiti III, M. flexor perforatus digiti IV and M. flexor hallucis brevis), with their separate origins and more proximal insertions on the interphalangeal joints, tend to provide more independent actions across the toes (figure 1). There have been few explanations of the relative roles of the proximally and distally inserted digit flexors in birds [11,23–25]. These explanations usually emphasize the roles of the superficial flexors in affording flexion of a particular toe independently of the others [25], or for positioning the interphalangeal joints to accommodate the sizes and shapes of grasped objects [11,23].

### 3.2 Mechanical analysis of grasping

There is a rich history of modelling digital tendon function in biomechanics, anthropology and medical bodies of literature since around the late 1960s, which extends well beyond our scope here. While these studies have generated great insights into the anatomical and biomechanical intricacies of the human hand, we seek a more general paradigm that covers avian foot musculoskeletal structure and function. Insights on the properties of underactuated mechanisms (UAMs) may shed light on the mechanical analysis of avian foot morphology and grasping capabilities. UAMs are kinematic systems in which there are fewer actuators than DOF. Multiple DOF may be actuated by a single actuator via a differential mechanism, allowing the system to passively adapt to contacts [26]. Principles of UAMs have been applied to robotic and prosthetic hand design, resulting in hands with few actuators that passively adapt to the grasped objects, thereby reducing the need for a complicated suite of sensors and control [26,27]. The actuation of these hands resembles the tendon actuation seen in birds' feet in that distally inserting tendons spanning multiple joints are used to actuate flexion of the digits [28–30]. Therefore, we believe that analysis techniques applied to their design may be used to investigate the relative roles of the proximally and distally inserted digital flexors, and help to explain how manipulative capabilities are preserved, and possibly enhanced, with relatively few muscles in birds.

**Figure 2.**
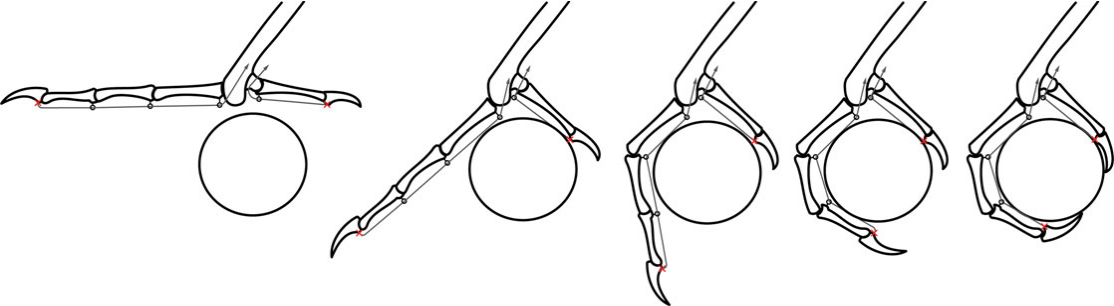
Diagram of how the digits wrap about an object, for a simulation of a single, distally inserted (red cross) tendon for digits I and III. For a given position of the object relative to the foot, the phalanges are sequentially wrapped about the object so that each link is tangent to the object and the talon tips make contact.

The complex emergent properties of bi- and multiarticular muscle systems and their advantages in comparison with monoarticular systems have been appreciated for some time [31,32]. For our purposes, multiarticular muscle systems may function like UAMs. A single tendon inserted on the distal phalanx of a digit and running over multiple interphalangeal joints acts to apply torques to all of those joints, causing the whole digit to flex. If the tendon does not have insertions on the more proximal phalanges, the joints can make contact (e.g. to wrap around a perch) without preventing continuing motion of the tendon and other phalanges (figure 2), and thereby imparting ‘passive adaptability’ to the digit. In this way, contact is enabled not by the action of multiple tendons/joints, but by the multiarticular routing of a single tendon. Aspects of this phenomenon have been observed in the digital flexor tendons of raptors [11] and in the digital extensor mechanism of mousebirds and parrots [33] as well as in humans [30,34]. If individuation of digital movement requires a greater number of muscles and neuromuscular coordination (e.g. [35]), then the potential reduction of musculature afforded by UAMs should be favoured in birds, for weight reduction, energetic savings and/or a more favourable concentration of muscle mass [36]. However, this may come at the cost of a reduction in the range of toe motions possible, and limited grasping capabilities [23].

In the past, robotics researchers have used birds both as source of inspiration for robotic mechanism design and as the basis for grasp analysis [37–39]. Our approach here differs fundamentally from these works in that we apply UAM principles and analytical techniques, instead, to inform aspects of avian hindlimb functional morphology that have long been subject to data deficiency and speculation. Accordingly, we provide an experimental context and new data to explore the potential causes and consequences of the observed variation in avian foot form and function.

## 4. Material and methods

Using a model to predict the performance of a grasping hand or foot is a notoriously difficult problem. The huge diversity of objects, initial object poses, and possible grasping poses and scenarios makes it infeasible to exhaustively model the behaviour. Because of this tractability problem, we are restricted to prototypical cases or object-agnostic proxies for performance such as the enclosed volume of a pre-grasp pose. In this paper, we present a model for understanding avian foot function in relation to morphology under two grasping conditions: perching and prey carriage. We measure this performance in terms of the ratio between the magnitude of the force applied to the foot (via either the weight of the bird (perching) or weight of the prey during carriage) and the summed total tendon tensions required to oppose it.

### 4.1 Foot model

We used a simplified two-dimensional foot model (after Backus *et al.* [27]) that captures the major characteristics of opposition in a grasping foot. However, we acknowledge that there is much greater complexity and variation in the nature of tendon insertions and interactions *in vivo* that are not accounted for in our model [22,40,41]. For our purposes, the foot model includes the tarsometatarso-phalangeal joint and distal elements of the first and third digits, as shown in figure 1. These digits tend to oppose one another in most anisodactylous (digits II–IV project cranially and digit I projects caudally) taxa [2]. Note that in many raptors, however, direct opposition of the hallux may instead be accommodated by digit II [19].

In the model, each digit is actuated by a flexor tendon inserted just distal to each inter- or distal (ungual) phalangeal joint (figure 1). Note that some of these tendons (e.g. M. flexor perforatus digiti III and M. flexor perforans et perforatus digiti III) branch at the point of insertion and attach to both distal and proximal ends (as well as the medial and lateral sides) of the joint capsule [22]. However, because the model cannot simulate multiple insertions, we approximated these by including a single tendon for each joint. Note that in doing so, we added an additional flexor not present in birds (corresponding to figure 1*a*) to represent the effect of M. flexor perforatus digiti III's proximal insertion on the D_3_P_1_. Lastly, we consider two possible actuation schemes: one where all tendons can exert forces independently on their phalanges of insertion, and another where only the distally inserted tendons are present.

### 4.2 Grasp pose

**Figure 3.**
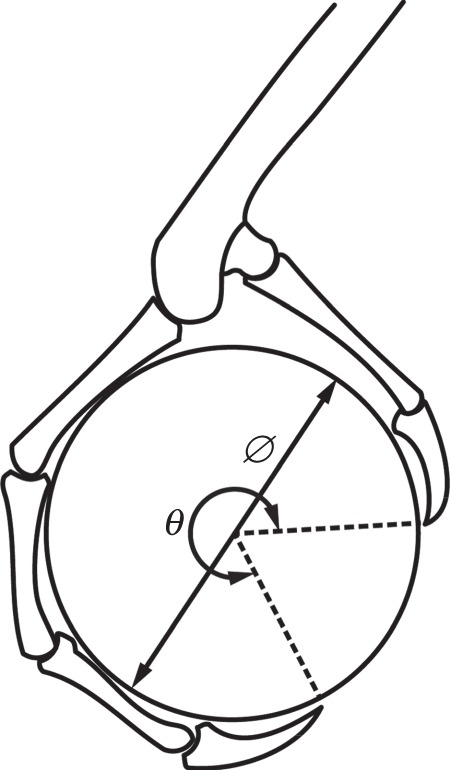
Diagram of an example grasp. The object is positioned relative to the tarsometatarsus to maximize the wrap angle while the individual phalanges are oriented tangent to the circular object. The wrap angle, *θ*, and object diameter, Ø, are overlain.

The joint angles, object position and contact locations, or collectively the ‘grasp pose’, are determined for each object size and set of phalanx lengths (hereafter referred to as the ‘kinematics’) as follows. First, we assume that the object can be approximated by a circle and that the links are straight line segments that are wrapped around the object so that each link is tangent to the circular object as shown in figure 2 [42]. We then find the object position relative to the tarsometatarsus that maximizes the angular wrap of the digits about the centre of the object as measured from the distal contact on one digit to the distal contact on the other (figure 3). Contacts between the object and digits are assumed to be at the points of tangency between them. We note that this process for defining the grasp does not consider the contact forces applied to the links and does not allow the grasp pose to change in response to the applied forces. Thus, the model assumes that all of the links are fixed in a particular pose, and finds the tendon forces that would keep them in static equilibrium. However, without this kind of simplification there is an infinite number of potential object/foot configurations, making the analysis intractable.

### 4.3 Force modelling

**Figure 4.**
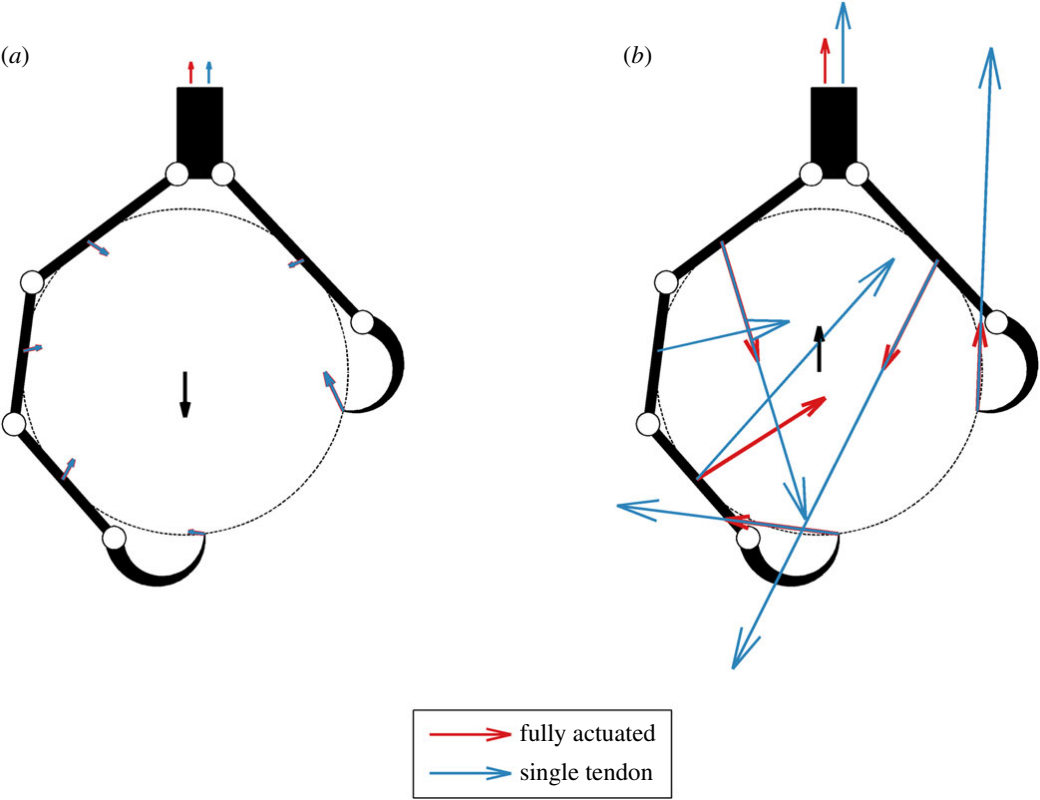
Diagrams showing the applied unit force vector (black) and resulting contact forces and total tendon tensions (indicated by the vectors drawn above the tarsometatarsus) when fully actuated (red) and actuated by a single tendon (blue) for a carrying grasp (*a*) and perching grasp (*b*). In all cases, the magnitudes of the contact force vectors and tendon tension vectors are drawn in proportion to the applied unit force vector and therefore all forces are unitless. In this example, there is an approximately 0% and approximately 57% difference in total tendon tension between single and fully actuated scenarios for the downward and upward force conditions, respectively. The latter difference reflects the relatively greater coupling of joint torques and contact forces when actuated by a single tendon, resulting in a relatively poorer performing single-tendon grasp that requires greater tendon forces than the fully actuated scenario.

Based upon the foot kinematics, grasp pose and contact locations, a relationship between a force applied to/by the object and the tendon forces needed to oppose it can be found. However, because the number of contacts between links and object over-determine the kinematics of the grasp, infinitely many possible solutions exist. We find the solution that minimizes contact and tendon forces by executing a least-squares minimization of contact forces and linear minimization of actuation forces. We arbitrarily set friction cone angles of 20^°^ for the proximal links and 80^°^ for the talon tip contacts, corresponding to friction coefficients of approximately 0.36 and 5.7, respectively. The friction coefficient for the proximal contacts (0.36) is within the range of many experimental results (0.2–0.7) and similar to what has been used in other simulations. We reasoned that the greater friction coefficient for the talon tip is assumed because the talon can dig into the grasped object, substantially increasing the shear force that it can resist (after Ramos & Walker [38]). However, instead of representing coulomb friction this corresponds to the talon tip shearing through the grasped object and the value may depend more on the material properties of the object and the shear forces it can resist. This minimization and associated constraints are implemented in matlab (v. 8.1.0.604) and solved using the quadprog function. This returns a plausible set of contact and actuator forces for a given force applied to the object. The single-tendon case may be analysed in the same manner by repeating the optimization with the additional constraint that the proximal tendon forces equal zero. Although our model can be used to investigate the response of a grasp to any force, we only consider upward and downward force directions, reflecting the forces the foot would experience when perching or grasping and lifting/carrying an object, respectively (figure 4).

### 4.4 Model parametrization and normalization

The model that we have proposed can be applied to any planar two-digit grasp. However, the large number of parameters that could be varied (link lengths, joint condyle radii, object diameter, force direction) results in an intractably large search space. Therefore, we have reduced the size of our parameter space based upon a number of assumptions. First, in order to eliminate the effect of variation in size among specimens, we normalized the phalanx lengths by the total span of the two digits (figure 1). Also, because of the limited number of complete datasets available (seven species, 11 specimens), the heights of the condyles of the tarsometatarso-phalangeal (TMT⋅P) and interphalangeal joints (P_proximal_⋅P_distal_; which we assumed to be proportional to the tendon moment arms at each joint) were not varied. Instead, we used the relationships between condyle heights [43] and direct measurements of tendon moment arms [17] versus digit lengths (electronic supplementary material, S1) to determine the following fixed moment arms (all with respect to the total foot length of 1): digit I joints: TMT⋅P_1_=0.04399, P_1_⋅P_2_=0.06995; digit III joints: TMT⋅P_1_=0.08111, P_1_⋅P_2_=0.05291, P_2_⋅P_3_=0.04156, P_3_⋅P_4_=0.05364.

Analysis of a more extensive dataset of digit I and III phalanx lengths compiled from the literature [2,17,43,44] shows that when normalized by the sum of D_3_P_1_, D_3_P_2_ and D_3_P_3_ lengths, the length of D_3_P_2_ does not vary significantly, while the relative lengths of D_3_P_1_ and D_3_P_3_ are inversely proportional (electronic supplementary material, figure S4). Based upon this analysis, we determined the following relationships for D_3_P_2_ and D_3_P_3_ given the other independent parameters:

4.1 $${\text{D}}_{3}{\text{P}}_{2}=0.3(1-\ell_{\mathrm{palm}}-{\text{D}}_{1}{\text{P}}_{1}-{\text{D}}_{1}{\text{P}}_{2}-{\text{D}}_{3}{\text{P}}_{4})$$

and

4.2 $${\text{D}}_{3}{\text{P}}_{3}=1-\ell_{\mathrm{palm}}-{\text{D}}_{1}{\text{P}}_{2}-{\text{D}}_{3}{\text{P}}_{1}-{\text{D}}_{3}{\text{P}}_{2}-{\text{D}}_{3}{\text{P}}_{4},$$

where ℓ_palm_ is the distance between the two proximal interphalangeal joints. Equation (4.1) indicates that the length of the second phalanx of digit 3 is equal to 0.3 times the total length of that digit (minus the length of the ungual), whereas equation (4.2) simply enforces that the total span of the foot is equal to 1. Lastly, statistical analysis of the talon lengths of digit I and III of all of the bird specimens we have data on suggests that there is no significant difference in the relative lengths of these two parameters (electronic supplementary material, figure S5).

Based upon these approximations, we modelled the fully actuated and single-tendon (underactuated) grasping capabilities of the 22 specimens (14 species) for which we found complete link length data (hereafter ‘bird-based’ kinematics; electronic supplementary material, table S2 and figure S6). We also examined a wide range of other potential kinematic combinations generated from the relationships we determined from available phalanx measurements, hereafter referred to as ‘parametrically generated’ kinematics. We explore the parameter space of possible foot configurations defined by the remaining free parameters (D_1_P_1_, D_3_P_1_ and talon length (D_1_P_2_, D_3_P_4_)), varying D_3_P_1_ and D_1_P_1_ from 0 to 1 and the talon length from 0 to 0.5 with a mesh spacing of 0.025. Each valid kinematic configuration was then simulated for objects ranging from 0.1 to 0.5 in diameter.

### 4.5 Grasp quality metric

The basic model we have implemented generates quantitative information about a grasp, namely the set of tendon forces and corresponding object contact forces that oppose the applied object force. To compare the capabilities of a single-tendon versus a fully actuated grasp, we simulate both cases and compare the tendon and contact forces (figure 4). To summarize the simulation results across all grasping scenarios, we also computed the per cent difference in total tendon tension between the single-tendon and fully actuated cases. A zero per cent difference indicates that the two grasps are equivalent in terms of total tendon force required. The greater the per cent difference, the lower the performance (i.e. the greater the tendon force required) of the single-tendon grasp relative to the fully actuated scenario.

### 4.6 Bird digit flexor proportions

Finally, we juxtaposed our simulation results with estimates of the relative sizes of the proximally and distally inserted digital flexor muscles among species. We calculated the proportion of muscle force (using *in situ* tension, muscle physiological or anatomical cross-sectional area (and muscle mass or relative volume, when the former two were unavailable)) for each digital flexor (relative to all flexors combined). We obtained these measurements from all published sources we could locate that reported quantitative data on the complete set of digit flexors for at least one specimen (electronic supplementary material, table S1). We restricted the dataset to those species that possess a functional hallux, as this digit is an integral component of our simulations. Although these data represent a small fraction of avian taxa comprising seven orders (Galliformes: Numididae; Falconiformes: Falconidae; Accipitriformes: Accipitridae, Cathartidae; Strigiformes: Strigidae, Tytonidae; Psittaciformes: Psittacidae; Passeriformes: Covidae, Strunidae; and Columbiformes: Columbidae), they span both ‘grasping/raptorial’ (mostly species that seize and carry prey with their feet) and ‘perching/walking’ (mostly vultures and non-predatory species that also spend considerable amounts of time on the ground) types of birds, across a range of body sizes.

## 5. Results

### 5.1 Simulation results

**Figure 5.**
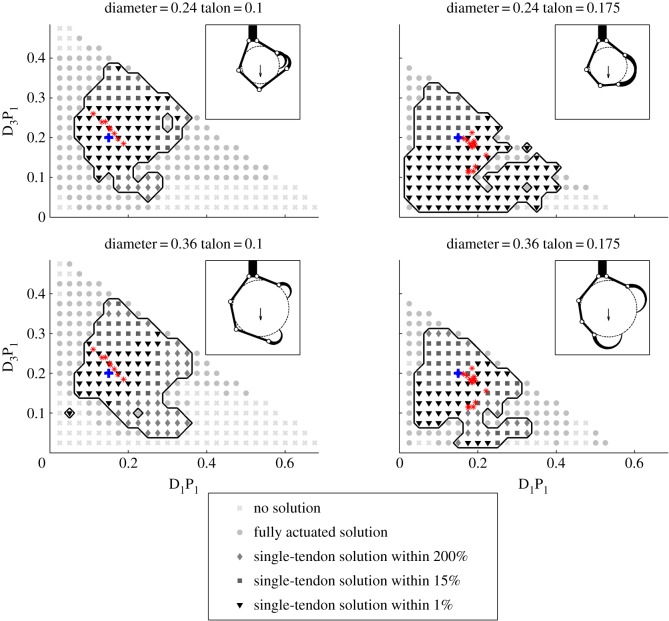
Parametric search results from simulations of digit I and digit III phalanx lengths, selected talon lengths and object diameters in response to a downward force. Plots show regions with different kinematic configurations where no solution (light grey cross), only a fully actuated solution (light grey dot), or a single-tendon solution exists for given kinematics. The region where both fully and single-tendon solutions exist is outlined with a solid line and further broken down by the per cent difference between solutions: within 1% (black triangle), 15% (dark grey square) and 200% (grey diamond). Lastly, the kinematic configurations of various actual birds (electronic supplementary material, figure S6) are plotted with red asterisks and the locations of the example grasps shown in the insets are plotted with a blue ‘+’ symbol.

**Figure 6.**
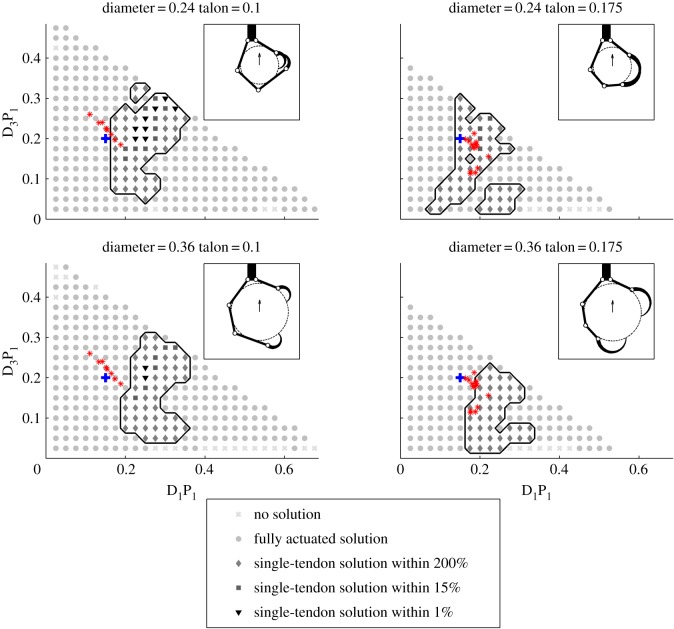
Parametric search results from simulations of digit I and digit III phalanx lengths, selected talon lengths, and object diameters in response to an upward force. Plots show regions with different kinematic configurations where no solution (light grey cross), only a fully actuated solution (light grey dot), or a single-tendon solution exists for given kinematics. The region where both fully actuated and single-tendon solutions exist is outlined with a solid line and further broken down by the per cent difference between solutions: within 1% (black triangle), 15% (dark grey square) and 200% (grey diamond). Lastly, the kinematic configurations of various actual birds are plotted with red asterisks and the locations of the example grasps shown in the insets are plotted with a blue ‘+’ symbol.

Figures 5 and 6 show slices of the parameter search space that we explored and highlight regions where single-tendon solutions will be found and others, generally with extremely long or short proximal link lengths, where none exist. Each subplot shows the range of proximal link lengths examined (all with respect to a total foot length of 1, and with the other parameters given via the equations and models described in §4) for a given talon length (0.1 or 0.175) and object diameter (0.24 or 0.36). For each parameter combination, the existence of a fully actuated and a single-tendon solution, as well as the per cent difference between them, is shown through the use of various symbols. Also, the normalized configurations of a number of actual birds (‘bird-based’) are mapped onto these plots (in red) according to their proximal link and average talon lengths. Figure 5 shows how the parameters affect the grasp when subject to a downward force representative of carrying an object. In this configuration, valid single-tendon and fully actuated grasps are found for most parametric configurations and the kinematics of actual birds' feet are near the centre of the region of valid single-tendon grasps. In comparison, figure 6 shows where single-tendon and fully actuated grasps may be found when an upward force is applied to the object when perching. In this scenario, fully actuated grasps exist for all but the most extreme kinematic combinations but single-tendon grasps only were found for a small range of D_1_P_1_ values. Also, unlike when grasping, in this situation most of the bird-based kinematics fall near the edge or completely outside the region where valid single-tendon solutions can be found.

**Figure 7.**
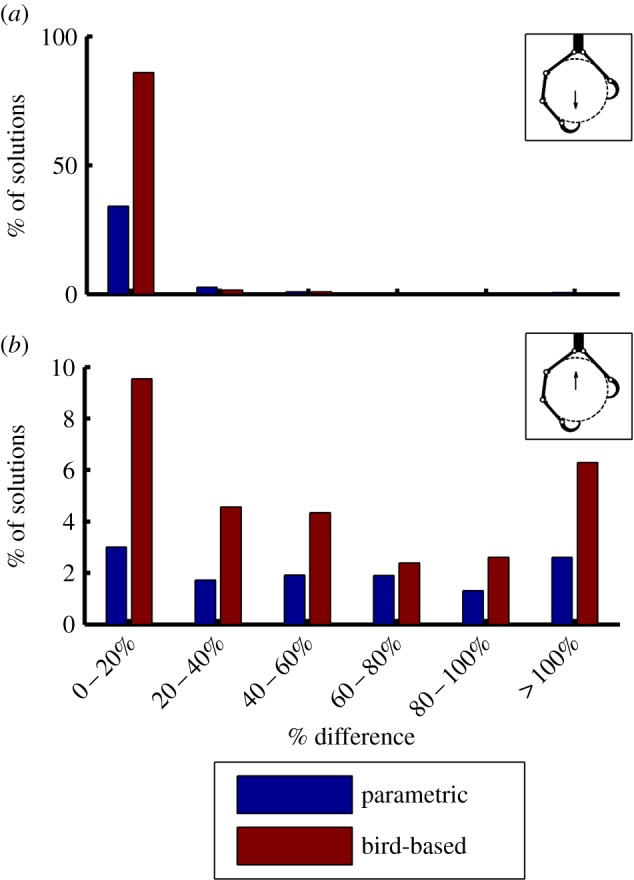
Summary of simulation results showing the performance of single-tendon grasps in response to downward (*a*) and upward (*b*) forces, for parametrically generated (blue) and bird-based (red) kinematic configurations. Insets in the top right of each subplot show an example grasp and applied object force. Each bar shows the percentage of single-tendon solutions within a certain range of per cent differences in total tendon tension between equivalent single-tendon and fully actuated grasps. The majority of kinematic configurations with single-tendon solutions performed nearly as well as their fully actuated counterparts when subjected to a downward force, while only a small fraction performed comparably for an upward force.

 Figure 7 summarizes the simulation results and shows the performance of single-tendon grasps in response to downward and upward forces, for parametrically generated and bird-based kinematic configurations. Each bar shows the percentage of single-tendon solutions within a certain range of per cent differences in total tendon tension between single tendon and fully actuated grasps based on the same kinematic configuration. The majority of kinematic configurations with single tendon solutions performed nearly as well as their fully actuated counterparts (i.e. within a 20% difference in performance) when subjected to a downward force (figure 7*a*). Conversely, for upward forces, the fraction of valid single-tendon grasps was considerably lower overall (figure 7*b*; note differences in scales between panels *a* and *b*), and the proportion of roughly equally performing single-tendon grasps was no greater than those that performed considerably worse (e.g. within a 40–100% difference in performance). These results also reveal that parametrically generated single-tendon grasps comprised a smaller percentage of the total number of valid solutions than did the bird-based kinematics. This discrepancy may be explained by the fact that the bird-based kinematics are clustered near the centre of the region spanned by the parametrically generated kinematics where solutions are most likely to be found, as can be seen in figures 5 and 6.

**Figure 8.**
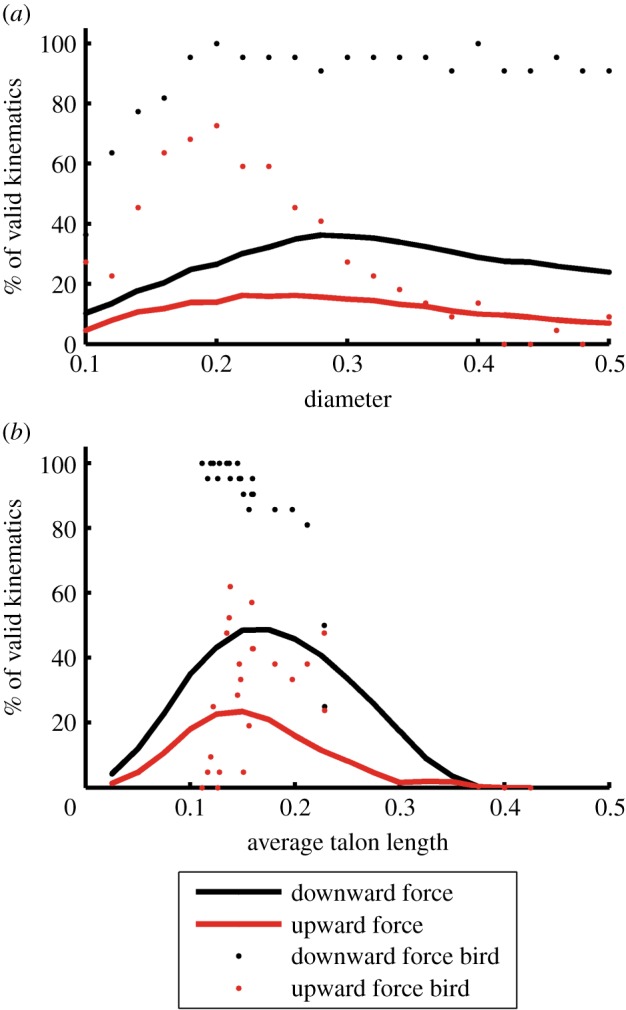
Percentage of grasps with a valid single-tendon solution as a function of (*a*) object diameter and (*b*) talon length (for the bird-based kinematics, the average of D_1_ and D_3_ talon lengths were used) for both upward (red) and downward (black) forces, for parametrically generated (solid lines) and bird-based kinematic configurations (‘bird’ points). The proportions of valid single-tendon grasps were larger for downward directed forces across all object diameters and talon lengths.

 Figure 8*a* demonstrates the effect of object diameter in terms of the percentage of grasps that have a single-tendon solution for each object diameter tested. For the parametrically generated grasps, single-tendon solutions were most likely to be found for objects around 0.24 for an upward force, and near 0.28 for a downward force (diameter/foot length), and the proportions of grasps with a single-tendon solution decreased for both larger and smaller diameters. Also, the difference in the percentages of grasps for upward and downward forces did not change substantially. Conversely, the bird-based kinematics (for the available species, most of which were raptors) demonstrated differences between downward and upward forces. When subjected to a downward force, single-tendon grasps existed for over 80% of all grasps based on bird kinematics, for objects greater than 0.15 in diameter. However, when subjected to an upward force, valid solutions existed for at least 60% of possible kinematics for grasps of objects between 0.15 and 0.21, but quickly dropped for larger diameters.

 Figure 8*b* shows the effect of talon length on the existence of single-tendon grasps, in terms of the percentage of grasps that have a valid single-tendon grasp as a function of average talon length. The results suggest that a talon length of between 0.125 and 0.175 is optimal for both upward and downward forces of parametrically generated grasps, and that a substantive increase or decrease in talon length is detrimental to single-tendon grasping performance. The bird-based kinematic results are much less clear. Talons of between 0.11 and 0.15 appear to be optimal in response to a downward force. However, no clear maximal region exists in response to an upward force.

### 5.2 Digital flexor muscle proportions

**Figure 9.**
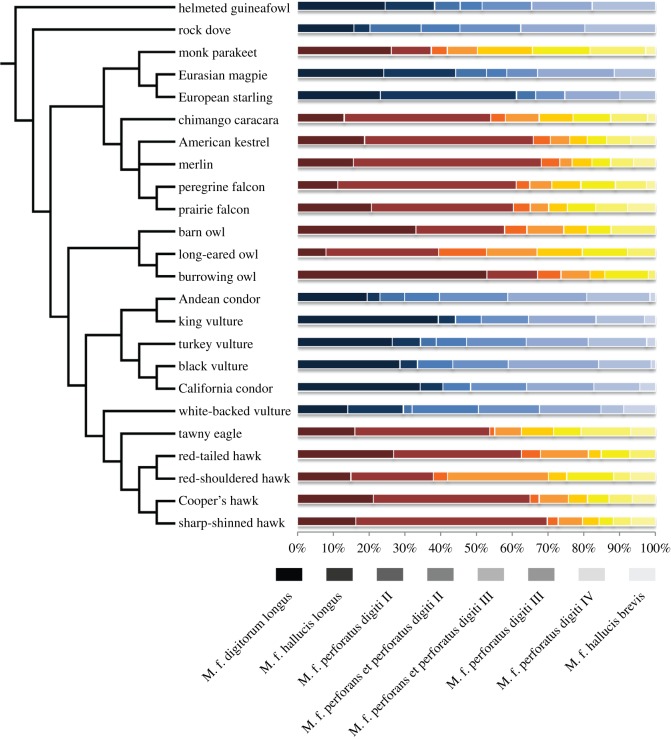
Distributions of proximally and distally inserted digital flexor muscles of avian taxa for which data were available (electronic supplementary material, S3, table S1). Values are based on *in situ* force, cross-sectional area, or mass (when the former were unavailable), averaged from multiple sources for each species, when available. ‘Grasping’ species are represented by the red–yellow bars, and ‘perching/walking’ species are represented by the blue–white bars. For both series, the first two darkest-coloured bars represent the distally inserted flexor musculature (Mm. flexores digitorum and hallucis longus), and the remaining lighter gradations of colour represent the proximally inserted flexors. Proportions of distally inserted muscles are significantly greater among raptorial, ‘grasping’ species that carry prey in their feet, whereas the proximally inserted flexors comprise greater proportions of digit flexor musculature in ‘perching/walking’ taxa. Phylogenetic topology was derived from Jetz *et al.* [45] (electronic supplementary material, S3).

The proportions of distally inserted flexor muscle force proxies (or mass) were significantly greater in grasping/raptorial species (*t*-test; *t*_22_=−3.63, *p*=0.001; electronic supplementary material, S3). Conversely, the proportions of proximally inserted flexor muscles were greater in those we classified as perching/walking (figure 9). Given the extent of phylogenetic clustering in our sampling, we cannot completely eliminate the role of historical contingency in driving these patterns. Nevertheless, the observed differences between groups held after adjusting for the effects of body size and phylogeny (OLS regression (through the origin) of independent contrasts of muscle proportions against grasp type; *r*^2^=0.23, *b*=0.19, *F*_1,21_=6.19, *p*=0.021; electronic supplementary material, S3 and figure S7).

## 6. Discussion

Previous researchers have theorized that reducing the size and number of muscles needed for grasping may provide a number of benefits. These include reduced neuromuscular coordination, overall reduction in muscle mass and energetic savings [35,36], but potentially come with a cost of reduction in grasping capabilities [23]. Our results (figures 5–9) suggest that, by extension, the elimination of all but the distally inserted flexors would not change the performance or force requirement of the grasp when applied to grasping and carrying tasks for most geometries, but are mostly not sufficient when the grasp supports the weight of the bird (e.g. during perching and walking). In the fully actuated case, when carrying an object, most of the tendon tension is applied through the distally inserted tendons, resulting in grasps similar to single-tendon grasps. However, when perching, the proximally inserted flexor tendons present in the fully actuated case can directly oppose the contact forces on the proximal links, resulting in grasp and tendon forces that differ greatly from the equivalent single-tendon grasps. An example of this is shown in figure 4: when grasping, the contact and tendon forces are identical for both the fully actuated and single-tendon cases. However when perching, the lack of the proximally inserting tendons in the single-tendon case results in significantly greater total tendon force and larger contact forces.

### 6.1 Distribution of hindlimb digit flexors

From our simulation, we show that birds may be able to perform grasping tasks using only distally inserted muscles under most circumstances with potentially little or no increase in required tendon force. Therefore, we expect that birds that perform these kinds of grasps regularly would dedicate a larger portion of their grasping musculature to the distally inserted tendons. The distribution of muscle forces (as approximated by cross-sectional area or mass) observed among taxa (figure 9) indeed suggests that species that are more likely to use their feet for grasping/carrying objects, such as the accipitrids, falconids and strigiforms (Strigidae, Tytonidae), tend to possess greater proportions of distally inserted digital flexor musculature (53–64%, on average). Conversely, those taxa that tend to grasp primarily in the context of perching, such as the passeriforms (Corvidae, Sturnidae), columbids and cathartids, tend to possess relatively greater proportions of proximally inserted muscles (39–80%, on average). Naturally, the distally inserted flexors of raptorial birds may be relatively hypertrophied simply for exerting greater force at the talons, for instance, to penetrate prey. However, previous work has suggested that talon penetration is not necessarily the primary mode of dispatching prey [14,18]. Nevertheless, the allocation of muscle force to the distal flexor tendons routed to the claws may be one way of enhancing overall grasping/prey carrying capability, in addition to claw force.

Although the muscle force distribution within taxonomic families described above is reasonably consistent, there are some outliers. The accipitrid vulture *Gyps africanus* is proportioned more like the New World cathartid vultures (i.e. with greater allocation to proximally inserted flexors), probably due to its similar ecology [46]. The unexpectedly low proportions of distally inserted digital flexors of *Buteo lineatus* compared with other accipitrids may relate to their preferences for relatively small prey [47]. Conversely, the European starling (*Sturnus vulgaris*) showed an unexpectedly high proportion of distally inserted digital flexor muscle mass, perhaps because two of the digital flexor muscles were omitted from Klijn's [48] dataset. In comparison with other species in our dataset, the monk parakeet (*Myiopsitta monachus*) exhibits greater dexterity in its feet for climbing and handling food [15,49], which may necessitate the greater development of the proximally inserted flexors [31]. We caution that the relative development of muscles is not a perfect indicator of tendon force distribution, since force, speed and energetic efficiency depend on a variety of architectural and physiological attributes of muscles and their associated tendons [50,51]. Thus, these, and other, taxa may demonstrate modifications in other dimensions of muscle physiology not considered here.

In addition to their apparent necessity for perching, the proximally inserted flexors may also play a role in more favourably distributing forces across the phalanges, perhaps to facilitate joint stability or postural movements of the toes. This effect partially supports Goslow's [11,24] suggestion that the proximally inserted flexors in raptors might counteract buckling at the interphalangeal joints resulting from the actions of the deep flexors inserted on the unguals, and thereby prevent loss of contact between toe and prey. Further support comes from Galton & Shepherd's [52] observations that starlings adjust their toe flexion to accommodate perch size.

### 6.2 Other kinematic factors

While many combinations of phalanx lengths are possible, the parametric simulation results suggest that feet with particularly long or short segments are much less likely to have single-tendon grasp solutions in the scenarios studied here. As can be seen in figure 5, the single-tendon solutions are most likely to exist for D_1_P_1_ lengths of between 0.075 and 0.325 and for D_3_P_1_ lengths of between 0.075 and 0.3 when grasping. By contrast, when perching, single-tendon solutions are most common for D_1_P_1_ lengths of 0.2–0.275 and D_3_P_1_ lengths of 0.075–0.2 as can be seen in figure 6. The parametric simulation results also suggest that valid single-tendon grasps are more likely for objects between 20 and 30% of total foot span for both upward and downward forces (figure 8*a*). This optimal range of diameters roughly corresponds to the digits completely wrapping around the object without overlapping considerably. Outside of this range, the portion of single tendon grasps decreases gradually for both grasping and perching. Additionally, the simulation results show the significance of talon length in opposing upward and downward forces in single-tendon grasps for the parametrically generated configurations. The simulations (figure 8*b*) suggested that mid-sized talons (approx. 15% foot span) were optimal, and performance dropped at both smaller and larger sizes. The characteristically large talons of hawks and eagles that hunt large prey [19] presumably have been selected for immobilizing [18], not necessarily carrying, large prey. Lastly, single-tendon solutions exist for a smaller set of these kinematic parameters when perching rather than grasping.

From the relatively sparse sampling of complete kinematic and musculo-tendon data from real birds that we had access to, it is interesting to note that the length of D_1_P_1_ fell within 11–22% of total foot span, the length of D_3_P_1_ within 11–26%, and the talon length within 8.7–23%. This means that all of the birds' link lengths are clustered near the centre of the region where single-tendon grasps are more likely to be found when grasping (figure 5). However, when perching (figure 6) the foot kinematics of some species fall outside of the region where single-tendon solutions are found due to the combination of a short D_1_P_1_ and talon. This suggests that some species may need to rely on the recruitment of proximally inserting flexors when perching.

Although generally consistent with the results from the parametrically generated configurations, the results in figures 7 and 8 for the bird-based kinematics are less clear. One contributing factor may be that the bird-based kinematic results were based on far fewer data points, comprising a very small fraction of avian taxa. Thus, any trends they suggest are not as conclusive as those from the parametrically generated grasps. Also, because the bird-based kinematics are clustered in a small region of the parameter space, trends they exhibit may be exaggerated. For example in figure 8*a*, a substantially greater portion of bird-based kinematics (in comparison with parametrically generated kinematics) have single-tendon solutions simply because the bird-based kinematics are clustered in the centre of the parameter space where single-tendon solutions are most likely to exist. Lastly, because we did not sample the full possible parameter space (but rather a reduced space in which only a few representative elements were varied for the sake of tractability), the bird-based kinematics do not map perfectly onto the parametric-generated kinematic space. Therefore, minor variations in other link parameters may account for the greater noise observed in the bird-based results.

### 6.3 Limitations and caveats

Our results suggest an equivalence in performance between single-tendon and fully actuated solutions for a given downward object force. Nevertheless, these actuation schemes do not preclude the existence of other solutions that may rely on other combinations of proximally and distally inserted tendons (e.g. [53]). Because the model ensures that a grasp is over determined, for any grasp pose and object force direction, there are many possible valid muscle force solutions. However, our analysis finds a muscle force solution that minimizes the sum of the tendon and contact forces. Also, since the model finds a minimum force solution for a particular pose and object force direction, predicted muscle force distributions can vary greatly for different object diameters and force directions. As a result, we cannot necessarily predict what flexor muscle proportions, or grasping performances, would look like simply from phalanx lengths, reflecting the ‘many to one’ mapping of morphology to performance phenomenon observed in many organismal study systems [54]. Nevertheless, we can determine whether or not a valid solution exists for a given set of muscle limitations, and with more information on how birds grasp we can further refine our model. These limitations may ultimately lend more practical insights, since natural selection does not operate by way of optimal search criteria, but rather incrementally, on existing variation.

Although we focused on two object force directions, corresponding to grasping and perching which we believe represent the primary forces that birds encounter while grasping, in reality birds will also encounter more complex object forces in other directions (e.g. when grasping struggling prey). These variable object force directions might be expected to alter the observed distribution of musculature. For instance, Fowler *et al.* [4,18] found that many raptors essentially stand atop their prey to immobilize them prior to feeding, which would ostensibly select for a more ‘perching’ muscle configuration (i.e. greater relative development of proximally inserted flexors). Indeed, our results show that the fully actuated solution performs substantially better than the single-tendon solution for upward-directed forces experienced during perching—and potentially prey immobilization—but importantly, that a single-tendon solution may still exist. This suggests that for some birds the advantages afforded by a foot that relies primarily on distally inserting tendons (for instance, to carry their quarry to a safer location for feeding) may outweigh the potential disadvantages for opposing other forces, such as those experienced during perching or immobilizing prey. Furthermore, initial explorations of how the muscle force required changes as a function of object force direction suggests that it changes smoothly for small changes in direction about the upward and downward directions we tested. Therefore, results should not differ dramatically for small changes in object force direction that would be expected during actual grasping and perching tasks.

Another significant limitation of our analysis is that we reduce the complex three-dimensional grasping problem to a planar two-dimensional case. Although this simplification is necessary to make the modelling tractable, it eliminates a number of object DOF that could cause a grasp to fail. These include the object translating in and out of the plane (as we have depicted it) as well as the object rotating about its secondary and tertiary axes. This simplification also means that our model does not include digits II and IV or consider the additional forces and constraints that they can impose. To the extent that these side toes ‘behave’ as do digits I and III in their respective planes, we do not anticipate that their inclusion should dramatically change our results, in terms of the disparity in performance between single-tendon and fully actuated grasps, and the relative distributions of proximally and distally inserted flexors. However, we cannot account for potential synergistic interactions that may occur among interdigital tendon and contact forces, and we reserve further speculation until such time that we can more accurately model them.

The model is also limited in how the grasp pose is defined and in that we do not allow the grasp to reconfigure in response to the applied forces. As described in §4.2, we define the foot configuration and contact locations based solely on the wrap angle metric. This simplification was necessary to ensure that grasps were defined in a consistent way but means that for some kinematics the resulting grasp may be far from optimal in terms of the tendon force required to resist the object force. In some extreme cases, no solution may be found simply because a poorly conditioned grasp pose was used. Similarly, we use the same grasp pose when analysing grasping and perching when in reality, the contact forces would cause the digits to reconfigure about the object and the resulting reconfiguration would depend on the object force direction. These simplifications of the model may explain some of the discontinuities observed in the parameter space. For example in figures 5 and 6, there are small regions where clusters of single-tendon solutions are separated from other such clusters by fully actuated and/or invalid solutions. Although it is tempting to assume that there is something unique about these regions, the kinematics in these regions when combined with the maximal wrap angle metric simply resulted in grasp poses that are poorly suited to single-tendon solutions.

Galton & Shepherd's [52] surgical intervention experiments of perched starlings may challenge some of our model assumptions and simplifications. For example, they suggested a ‘very small contribution by the flexor tendons during unstressed perching’ [52]. Thus, the roles of body posture and balance during perching may be underestimated here. However, it is quite likely that, under more stochastic grasping conditions (e.g. perching in inclement weather and/or grasping struggling prey), the forces considered here, and various others, need to be more actively opposed by the digital flexor muscles and tendons. These authors also observed that perched starlings shifted their weight over the central foot pad, which rested directly on the perch during sleeping [52]. Our model did not predict palmar contact for the kinematics we considered (i.e. direct contact with the distal end of the tarsometatarsus), which could have substantially altered the predicted performance of grasps for upward forces [27]. However, the area of the distal tarsometatarsus between the phalanges is very small and padded with thick layers of fat, fascia and skin that may distribute contact forces over a broader area [55]. Therefore, the extent to which birds rely on this (and only this) surface for weight-bearing is unclear.

## 7. Conclusion and implications

**Figure 10.**
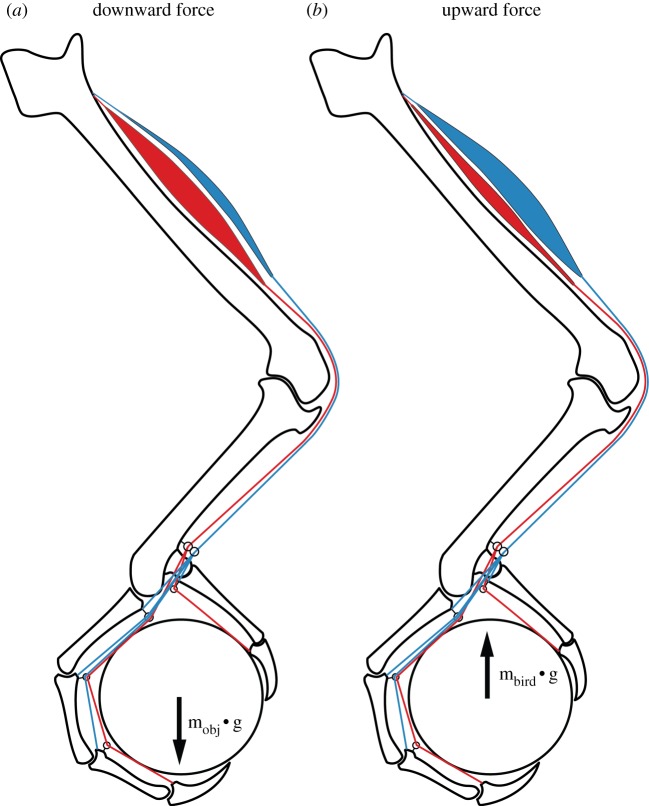
Summary of the main findings of the paper. Our simulation results suggest that, for a given amount of total muscle force, the distally inserted flexors (e.g. Mm. flexors digitorum and hallucis longus; red) perform just as well for carrying objects (*a*), as when all flexors are used. Conversely, when perching (*b*), the distally inserted tendons, alone, are insufficient, and the proximally inserted flexors (e.g. M. flexor perforatus group; blue) play a greater role in generating the requisite forces. These trends are reflected in the relative development of these sets of muscles: the ratio of distally to proximally inserted digit flexors is larger in birds that typically grasp and carry objects. Refer to figure 4 for a more thorough depiction of relative tendon and contact forces under these grasping scenarios.

Our results demonstrate the impact of various kinematic and grasp parameters, including object diameter and relative link lengths, on the relative performance of fully actuated and single-tendon grasps when downward and upward-directed forces are applied. Given the assumptions of our model and analytical constraints, we show how a single tendon is sufficient for most grasping tasks, but is often insufficient for perching tasks. All of the birds for which we found data are effectively fully actuated, potentially because the additional muscles are needed to stabilize grasped objects in ways that our model does not consider. Even though all of these flexors are present in the taxa we studied, the relative distribution of their muscles varies significantly. Birds that rely on grasping and carrying their prey (e.g. hawks and falcons) tend to have a greater proportion of their musculature allocated to the distally inserted digital flexors, whereas in other non-raptorial birds (e.g. passeriforms and vultures) proximally inserted flexors comprise a greater proportion of their musculature (figure 10).

These results may help our understanding of distal hindlimb muscle evolution and function in birds. Several modifications in the numbers, sizes, and attachments of the foot muscles have evolved in the lineage of theropods leading to modern birds [56], as well as within modern avian lineages [57]. For instance, while the vast majority of avian lineages have retained all of the extrinsic muscles, some groups (e.g. Passeriformes, Coraciiformes (kingfishers) and Piciformes (woodpeckers)) have lost variable numbers of the intrinsic foretoe muscles [22,23]. Our results suggest that these complex patterns of gains, losses and modifications may be correlated with evolutionary trends in phalanx length proportions [2,43]. However, these correlations may only emerge, and consequently be understood, in an integrative functional context supplied by a simulation framework that facilitates assessment of the relative performances of all possible kinematic configurations [34]. Naturally, these configurations are limited by parametric simplifications and data limitations. Thus, future approaches that encompass larger search spaces, parametrized by muscle data on a greater diversity of species—particularly passeriforms—are certainly warranted.

## Supplementary Material

Supplementary Materials: Mechanical analysis of avian feet: multiarticular muscles in grasping and perching - Document detailing the parameter reduction we performed on the measurements of specimens, the results of which were used as the basis for our parameter search.

## Supplementary Material

Supplementary Materials Tables - Table of the aggregated link length and muscle force data used throughout the paper.

## Supplementary Material

Simulation results - file containing the parameterization and raw output from out simulation.

## Data accessibility

Data used to parametrize simulations and raw simulation results have been included in the electronic supplementary material associated with this paper.
