# Supplementary material for: Mechanical analysis of avian feet: multiarticular muscles in grasping and perching
Source: R Soc Open Sci. 2015 Feb 25;2(2):140350. doi: 10.1098/rsos.140350 (PMC4448815; doi:10.1098/rsos.140350)
Supplement: Supplementary Materials: Mechanical analysis of avian feet: multiarticular muscles in grasping and perching - Document detailing the parameter reduction we performed on the measurements of specimens, the results of which were used as the basis for our parameter search. [file rsos140350supp1.pdf]

Supplementary materials: Mechanical analysis of avian feet: multiarticular muscles in grasping and perching

Spencer B. Backus[1,*], Diego Sustaita[2], Lael U. Odhner[1] and Aaron M. Dollar[1]

[1]*Department of Mechanical Engineering and Materials Science, Yale University, New Haven, CT 06511 USA*
[2]*Department of Ecology & Evolutionary Biology, Brown University, Providence, RI 02917 USA*

S1. Analysis and parameterization of interphalangeal joint condyle heights

We used a combination of two data sets to derive estimates of joint condyle heights, which formulate the interphalangeal joint moment arms in our models. These data sets were generated from (1) direct measurements of the tendon moment arm of falcons and accipiter hawks (measured in mm from the estimated joint center of rotation to the arc followed by the tendon [1]), and (2) skeletal measurements of the heights (mm) of both the proximal and distal condyles of the first three phalanges of digit III across a much wider range of avian taxa [2]. We found that P1-P3 condyle heights [2] and the measured tendon moment arms of P1-P4 [1] varied linearly with digit III length (mm; approximated by the sum of the lengths of P1-P3). These positive relationships reflect that larger feet exhibit condyles and tendon moment arms of greater height. Furthermore we found that the difference in slopes between Sustaita's [1] tendon moment arms and half the height (i.e., the radius; an approximation of the perpendicular distance between the joint center of rotation and the line of tendon action) of Kambic's [2] distal condyle measurements of the phalanx proximal to the joint is statistically insignificant, for the second (P1·P2) and third (P2·P3) joint. Therefore we assumed that the slopes of the regressions of the tendon moment arms for the first (TMT·P1) and fourth (P3·P4) joints [1] , as well as the slopes of the regressions derived from the combined tendon moment arms [1] and the heights of the condyles proximal to the second and third joints [2], correspond to the relative joint moment arms in most birds. Both the data used in these regressions and the regressions themselves are shown in figures S2 and S3.

Based upon this analysis we found the following slopes for the moment arm-digit length relationships: Digit I: $TMT \cdot P1 = 0.04399$ $(R^2 = 0.95, F_{1,10} = 227.8, P < 0.001)$, $P1 \cdot P2 = 0.06995$ $(R^2 = 0.96, F_{1,10} = 292.8, P < 0.001)$; Digit III: $TMT \cdot P1 = 0.08111$ $(R^2 = 0.98, F_{1,10} = 505, P < 0.001)$, $P1 \cdot P2 = 0.052914$ $(R^2 = 0.93, F_{1,144} = 2021, P < 0.001)$, $P2 \cdot P3 = 0.041562$ $(R^2 = 0.93, F_{1,144} = 1963, P < 0.001)$, $P3 \cdot P4 = 0.05364$ $(R^2 = 0.95\ F_{1,10} = 219.4, P < 0.001)$ (figures S2, S3). Thus, for each phalanx in our in our simulations we assigned a condyle height of a constant fraction (slope) of digit length. We recognize that the observed (linear) variation in condyle heights can affect the tension required to oppose upward and downward disturbance forces, and thereby alter the simulation results. Nevertheless, we chose not to vary condyle heights, and instead use the set values described above, because our primary objective was to model the effects of link (phalanx) lengths; were we to vary condyle heights, these effects would have been conflated with those of variation in the total lengths of digits I and III. In addition, because the relationship is linear, the effects would likely be a matter of scale, such that longer digit III lengths would systematically reduce the tendon tension requirement (due to their relatively larger condyles). Although this reduction may alter the landscape of valid solutions, the relative performances of fully actuated and single-tendon configurations - the main focus of our simulations - would likely remain unaffected.

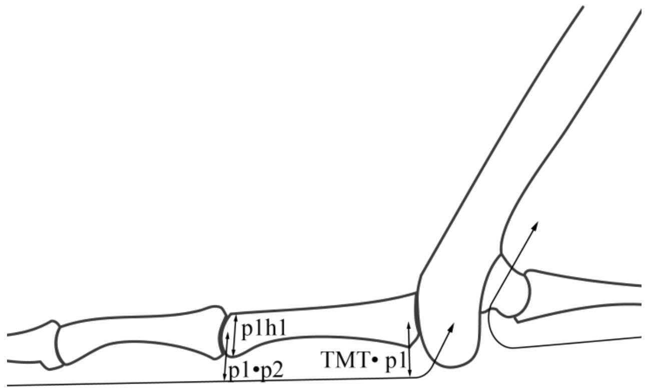

Figure S1. Diagram of TMT and proximal phalanx showing the tendon moment arm measurement for the TMT·P1 joint and both the tendon moment arms (P1·P2) and the height of the distal condyle (P1H1) proximal to the joint. When modeling the P1·P2 joint, we used both ½ the height of the distal condyle proximal to the joint (P1H1) and the measurement from the joint center of rotation to the tendon (P1·P2).

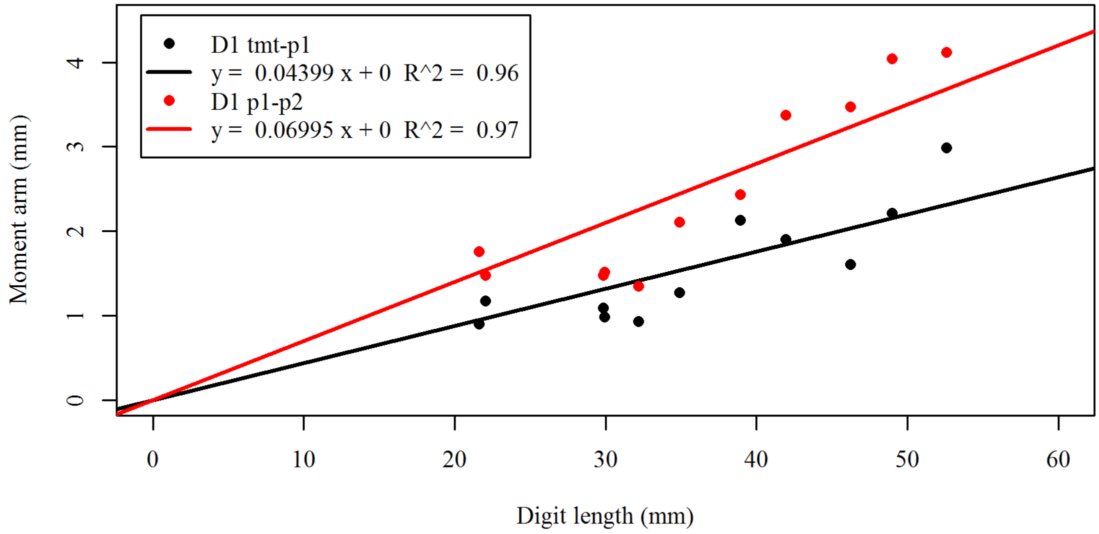

Figure S2. Digit I interphalangeal joint condyle heights (as proxies for tendon moment arms) versus digit I length.

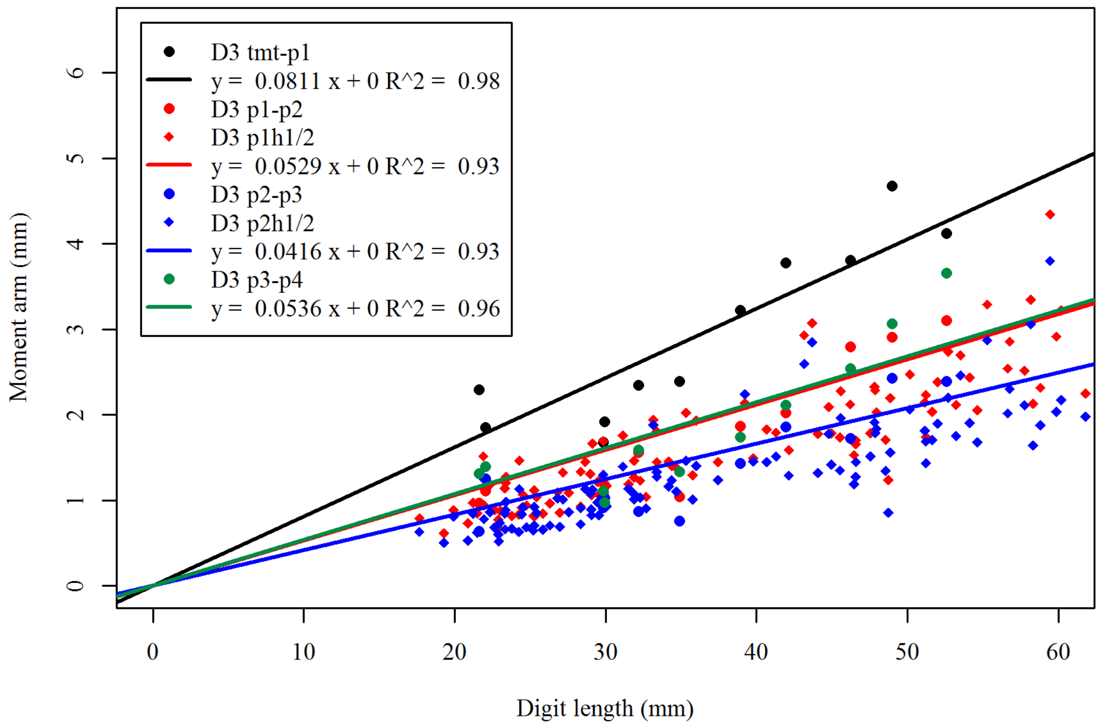

Figure S3. Digit III interphalangeal joint condyle heights (as proxies for tendon moment arms) versus digit III length.

S2. *Analysis of normalized digits I and III phalanx lengths*

In order to reduce the parameter space we explored, we analyzed measurements of the proximal phalanx of digit III [2, 3] as well as measurements of all of the phalanx and talon lengths of digits I and III [1, 4]. To allow for meaningful comparisons among specimens, all lengths were normalized by the total length of the first three phalanges of digit III. Analysis of the normalized phalanx lengths of digits I and III shows two statistically significant pairwise relationships between phalanx lengths and one invariant phalanx length. First, the length of digit III phalanx 2 (D3P2) does not depend on digit III phalanx 1 (D3P1) and is therefore assumed to be constant (approximately 0.3 of total digit III length; figure S4). Second, there is a clear inverse relationship between D3P1 and digit III phalanx 3 (D3P3); $D_3P_3 = -0.96D_3P_1 + 0.68$ ($R^2 = 0.68, F_{1,344} = 725.4, P < 0.0001$). We would expect to see this relationship based on the normalization and invariant nature of D3P2 and therefore round to $D_3P_3 = -D_3P_1 + 0.7$ (figure S4). These parameter reductions are consistent with Kavanagh et al.'s [5] findings for digit IV within and among avian species, in that the size of a given phalanx can be predicted from the sizes of the other two. Lastly, the lengths of the ungual phalanx (including the claw) of the first (D1P2) and third (D3P4) digit are effectively equal ($R^2 = 0.96$, $F_{1,21} = 475.1$, $P < 0.0001$; figure S5). To summarize, we use the following three relationships: $D_3P_2 = 0.3(D_3P_1 + D_3P_2 + D_3P_3)$, $D_3P_3 = -D_3P_1 + 0.7$, and $D_1P_2 = D_3P_4$ to reduce the number of parameters needed to describe the foot kinematics, from six to three.

Furthermore, in order to ensure consistent overall foot geometries, the total span of the foot, from the distal extent of D1P2 to that of D3P4, was normalized to 1. With this normalization, the relationships can be re-expressed as follows: $D_1P_2 = D_3P_4$; $D_3P_2 = 0.3(1 - \ell_{palm} - D_1P_1 - D_1P_2 - D_3P_4)$; and $D_3P_3 = 1 - \ell_{palm} - D_1P_1 - D_1P_2 - D_3P_1 - D_3P_2 - D_3P_4$.

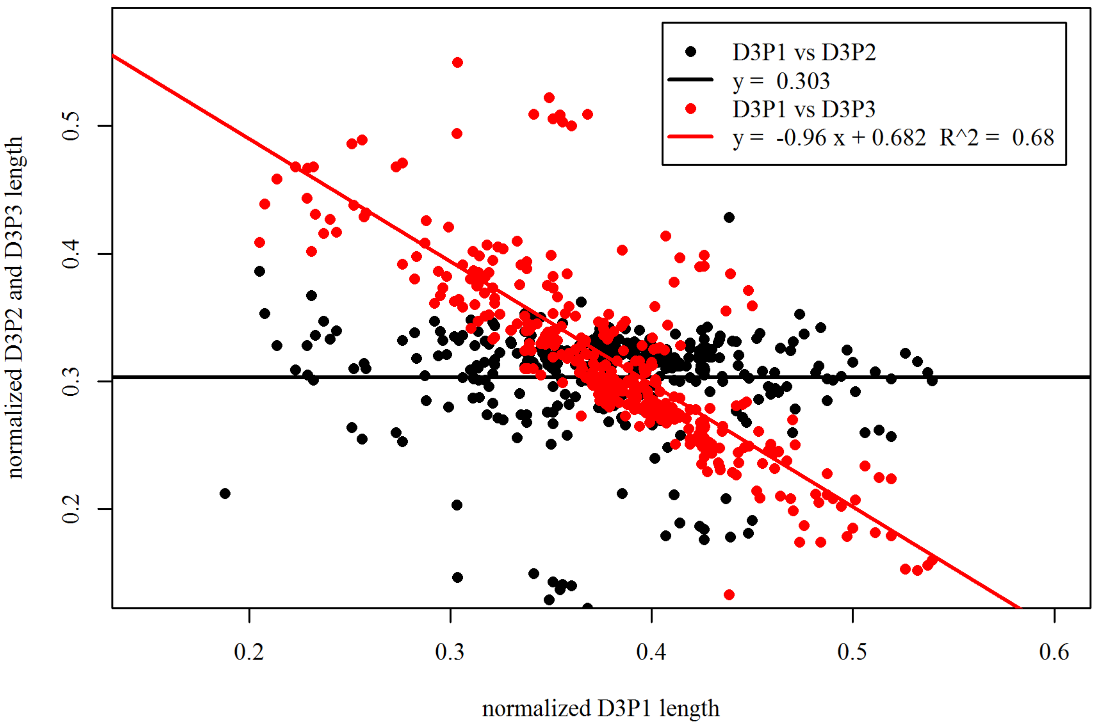

Figure S4. Lengths of D3P2 and D3P3 with respect to the length of D3P1, normalized by the sum of D3P1-D3P3. These data show that (1) there is no statistically significant relationship between the lengths of D3P2 with respect to D3P1, and (2) the inverse relationship between D3P3 and D3P1.

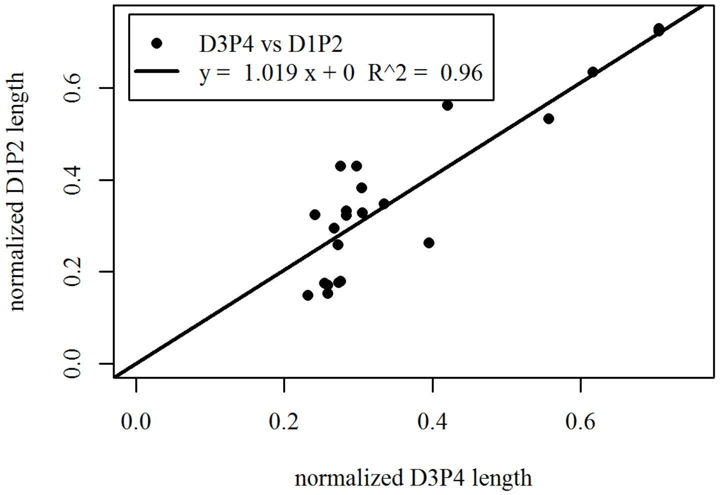

Figure S5. Length of D1P2 with respect to the length of D3P4, normalized by the sum of D1P1-D1P2, demonstrating that, based upon 22 specimens, the lengths of digit I and III ungual phalanges (including the claws) are approximately equal.

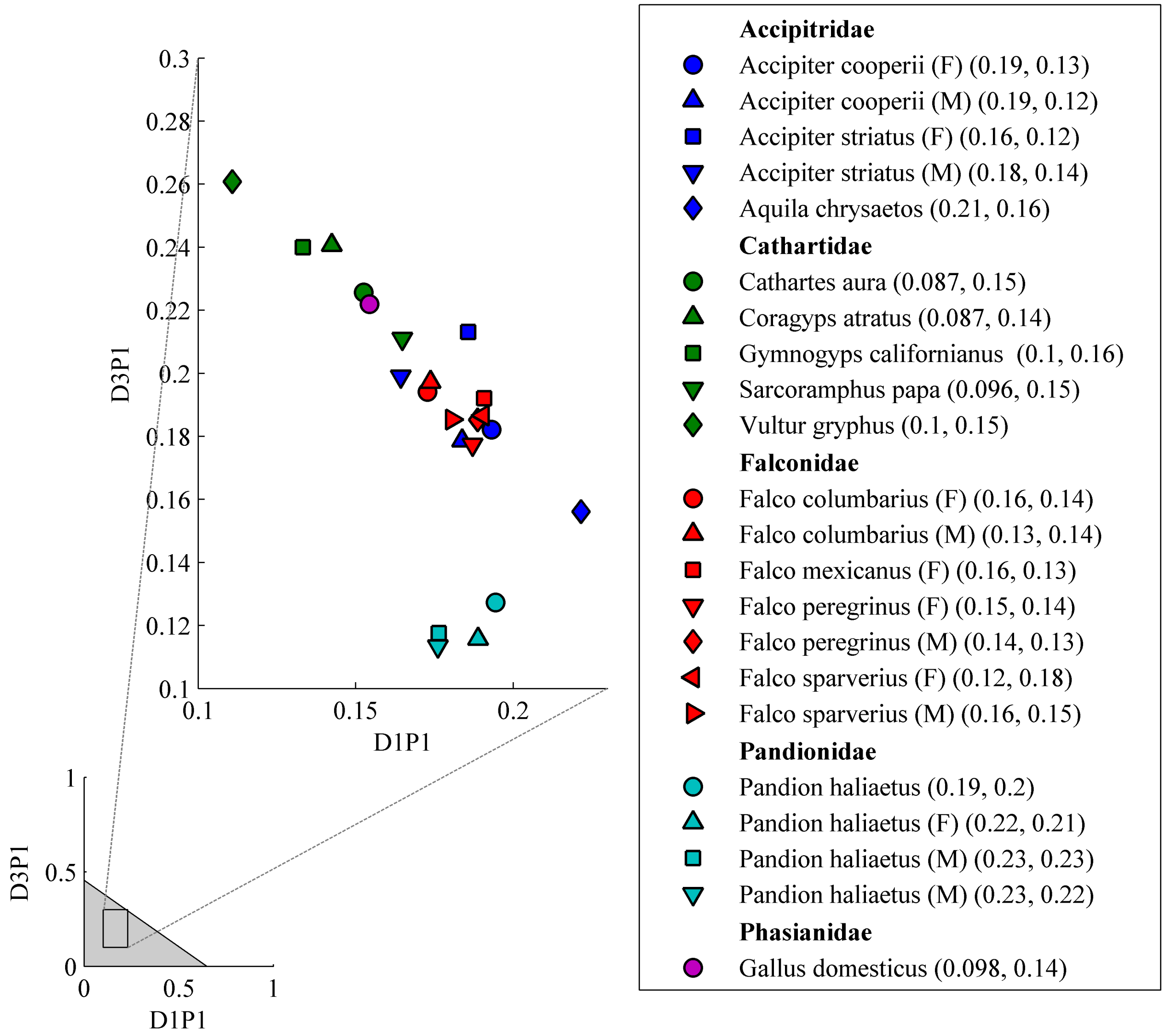

Figure S6. Bivariate space of the first phalanges of digits I and III, showing the kinematic configuration of each specimen (“bird-based kinematics”) used in the modeling simulations described in the text.

S3. *Analysis of digit flexor muscle proportions*

We compiled proportions of distally-inserted (deep) and proximally-inserted (superficial and M. flexor hallucis brevis) digital flexor muscle mass, cross-sectional area, or *in situ* tension (to total digital flexor musculature) for various species from the literature (Table S1) [1, 4, 6-11]. We also tabulated body mass when available for each species studied by each source; otherwise we obtained estimates from the literature. We averaged the proportions for those species that contained data from separate sources to test for differences between ‘grasping’ and ‘perching’ species, based on our coarse-grained assessments of their general behavior. We also performed additional analyses on recalculated muscle proportions that excluded M. flexor hallucis brevis (MFHB), since some sources did not include this muscle. However, since the results of these latter analyses did not differ, we report data and analyses including MFHB.

We used phylogenetically-independent contrasts (PIC) to account for phylogenetic relationships among the species in our data set, according to methods described by Garland et al. [12, 13]. We sampled 10,000 “phylogeny subsets” of the 24 species in our data set (Table S1) from Jetz et al.’s [14] complete bird phylogeny, from http://birdtree.org/ (using the “Hackett All Species” option). We then used MESQUITE [15] to estimate a consensus

tree topology (majority rules, required frequency of clades = 0.5, trees treated as rooted), primarily for graphical purposes (figure 9). However, we also used this consensus tree (with [ln] ultrametric branch lengths) to generate standardized PICs of deep flexor muscle proportions and grasp type (dummy coded; 1 = grasping/raptorial birds, 0 = perching/walking birds), and performed an ordinary least squares (OLS) regression through the origin using the PDAP:PDTREE module [16] (figure S7). The effect of body mass on the distribution of flexor musculature was not significant after adjusting for phylogeny, and therefore we did not incorporate body mass in the analysis. To determine the significance of the regression, we reduced the degrees of freedom by 1 due to a polytomy in the consensus tree [17]. To verify the robustness of the PIC regression based on the consensus tree, we created 100 additional phylogeny subsets of the 24 species, based on each one of the four "backbone" source phylogeny options provided in http://birdtree.org/, and ran separate PIC OLS regressions for every tree (using their original branch lengths). Collectively, across all 400 regressions, r^2 ranged 0.16 – 0.24 and p-values ranged 0.016 – 0.054 (only one of 400 regressions resulted in $p \geq 0.05$).

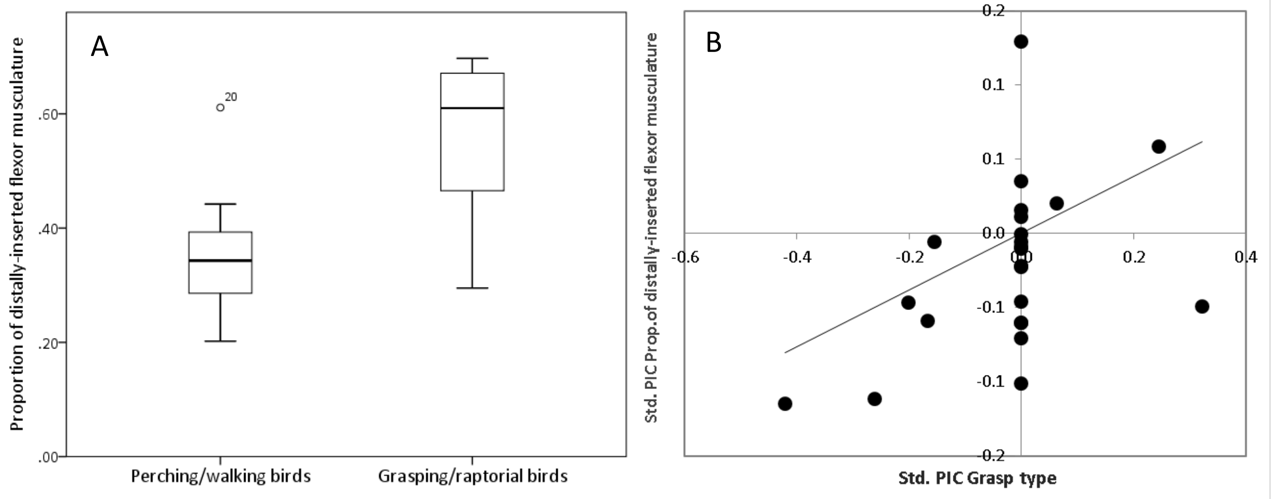

Figure S7. (A) Raw proportions of distally-inserted digital flexor muscle (M. flexors hallucis and digitorum longus) mass, cross-sectional area, or in situ tension relative to total proximally- and distally-inserted digit flexor musculature combined, for various grasping/raptorial (*n* = 15) and perching/walking (*n* =9) species, compiled from various sources. (B) Standardized phylogenetically-independent contrasts (Std. PICs; *n* = 23) of the proportion of deep flexors vs. Std. PICs of grasp type, based on a consensus tree of the 24 species in our sample. The significant (positive) relationship (r^2 = 0.23, $F_{1,21}$= 6.19, *P* = 0.021) indicates that the proportions of distally-inserted digital flexors are greater among species classified as grasping/raptorial birds. (The results for the proportions of proximally-inserted flexors are essentially the reciprocals of those depicted in A and B).

REFERENCES

[1] Sustaita, D. 2008 Musculoskeletal underpinnings to differences in killing behavior between North American accipiters (Falconiformes: Accipitridae) and falcons (Falconidae). *Journal of Morphology* **269**, 283-301.

[2] Kambic, R.E. 2008 Multivariate analysis of avian and non-avian theropod pedal phalanges. Bozeman, Montana, Montana State University.

[3] Hopson, J.A. 2001 Ecomorphology of avian and nonavian theropod phalangeal proportions: implications for the arboreal versus terrestrial origin of bird flight. In *New Perspectives on the Origin and Early Evolution of Birds: Proceedings of the International Symposium in Honor of John H. Ostrom* (eds. J. Gauthier & L.F. Gall), pp. 211-235. New Haven, Yale University.

[4] Fisher, H.I. 1946 Adaptations and comparative anatomy of the locomotor apparatus of New World Vultures. *The American Midland Naturalist* **35**, 545-727.

[5] Kavanagh, K.D., Shoval, O., Winslow, B.B., Alon, U., Leary, B.P., Kan, A. & Tabin, C.J. 2013 Developmental bias in the evolution of phalanges. *Proceedings of the National Academy of Sciences* **110**, 18190-18195. (doi:10.1073/pnas.1315213110).
[6] Goslow, G. 1967 Functional Analysis of the Striking Mechanisms of Raptorial Birds, University of California, Davis.
[7] Hertel, F., Maldonado, J.E. & Sustaita, D. in press Wing and hindlimb myology of vultures and raptors (Accipitriformes) in relation to locomotion and foraging. *Acta Zoologica (Stockholm)*.
[8] Klijn, H.B. 1976 An analysis of muscle weight variations in the wing and leg of Sturnus vulgaris. *Anat Embryol (Berl)* **149**, 259--287.
[9] Mosto, M.C., Carril, J. & Picasso, M.B. 2013 The hindlimb myology of Milvago chimango (Polyborinae, Falconidae). *J Morphol* **274**, 1191-1201. (doi:10.1002/jmor.20172).
[10] Verstappen, M., Aerts, P. & Vree, F.D. 1998 Functional morphology of the hindlimb musculature of the black-billed magpie, *Pica pica* (Aves, Corvidae). *Zoomorphology* **118**, 207-223.
[11] Carril, J., Mosto, M.C., Picasso, M.B. & Tambussi, C.P. 2014 Hindlimb myology of the monk parakeet (Aves, Psittaciformes). *J Morphol*. (doi:10.1002/jmor.20253).
[12] Garland, T., Jr., Harvey, P.H. & Ives, A.R. 1992 Procedures for the analysis of comparative data using phylogenetically independent contrasts. *Systematic Biology* **41**, 18-32.
[13] Garland, T., Dickerman, A.W., Janis, C.M. & Jones, J.A. 1993 Phylogenetic Analysis of Covariance by Computer Simulation. *Systematic Biology* **42**, 265-292. (doi:10.1093/sysbio/42.3.265).
[14] Jetz, W., Thomas, G.H., Joy, J.B., Hartmann, K. & Mooers, A.O. 2012 The global diversity of birds in space and time. *Nature* **491**, 444-448. (doi:10.1038/nature11631).
[15] Maddison, W.P. & Maddison, D.R. 2011 Mesquite: a modular system for evolutionary analysis. (2.75 ed.
[16] Midford, P.E., T. Garland, J. & Maddison, W. 2011 PDAP:PDTREE package for Mesquite. (1.16 ed.
[17] Purvis, A. & Garland, T.J. 1993 Polytomies in comparative analyses of continuous characters. *Systematic Biology* **42**, 569-575.
